# Functional Block Copolymers Carrying One Double-Stranded Ladderphane and One Single-Stranded Block in a Facile Metathesis Cyclopolymerization Procedure

**DOI:** 10.3390/ijms20205166

**Published:** 2019-10-18

**Authors:** Wei Song, Yadi Li, Xunhu Liu, Zongyi Xu, Jianhua Wu, Liang Ding

**Affiliations:** 1 Department of Polymer and Composite Material, School of Materials Engineering, Yancheng Institute of Technology, Yancheng 224051, China; bhf12345@126.com (Y.L.); 19851543862@139.com (X.L.); xzy9812@163.com (Z.X.); 2 Department of Materials, College of Physics, Mechanical and Electrical Engineering, Jishou University, Jishou 416000, China

**Keywords:** metathesis cyclopolymerization, spectroscopy, ladderphane, perylene diimide

## Abstract

In order to improve the poor film-forming ability of polymeric ladderphane, di-block copolymers containing perylene diimide (PDI)-linked double-stranded poly(1,6–heptadiyne) ladderphane and branched alkyl side chains modified single-stranded poly(1,6–heptadiyne) were synthesized by metathesis cyclopolymerization (MCP) using Grubbs third-generation catalyst (Ru–III) in tetrahydrofuran solvent. The first block containing the ladderphane structure leads to higher thermal-stability, wider UV–vis absorption, lower LUMO level and ladderphane-induced rigidity and poor film-forming ability. The second block containing long alkyl chains is crucial for the guarantee of excellent film-forming ability. By comparing the effect of ladderphane structure on the resulted copolymers, single-stranded poly(1,6–heptadiyne) derivatives with PDI pedant were also processed. The structures of copolymers were proved by ^1^H NMR and gel permeation chromatography, electrochemical, photophysical, and thermal-stability performance were achieved by cyclic voltammetry (CV), UV-visible spectroscopy and thermogravimetric analysis (TGA) measurements. According to the experiment results, both copolymers possessed outstanding film-forming ability, which cannot be realized by small PDI molecules and oligomers. And they can serve as a superior candidate as for n-type materials, especially for their relatively wide range of light absorption (λ = 200~800 nm), and lower LUMO level (−4.3 and −4.0 eV).

## 1. Introduction

It is well-known that perylene diimide (PDI) chromophores are widely used for constructing n-type organic semiconductors, as PDI derivatives generally show high thermal and chemical stabilities, good electron-accepting abilities, and excellent electron mobilities [1,2,3,4,5]. Compared to fullerene derivatives, the superior light-absorbing strength of PDIs in the visible range can be extended to 400-600 nm, depending on the electron-donating or electron-accepting substitutes on the bay positions. However, most PDI molecules possess strong crystallizability, which will lead to wide phase separations, reduced exciton diffusion/separation efficiencies, and finally, low power conversion efficiency of the organic solar cells [6].

Great progress has been made on a new class of polymers, which was defined as polymeric ladderphane comprising of double stranded polymeric skeleton with multilayer planar oligoaryl linkers. More precisely, a linear array of co-facially oriented arenes with polymeric backbones as the tethers [7,8,9,10]. In addition, the space between neighboring linkers is nearly 0.4 nm, similar to the distance of π–π stacking, which will facilitate electron-delocalization and enhance conjugation degree [11]. Along with these fast advances in polybinorbornene-based ladderphanes, many efforts have also been devoted to exploring the ladderphanes consisting of non-polybinorbornene skeletons. A novel double-stranded polyacetylene ladderphane with a PDI bridge has been efficiently synthesized by metathesis cyclopolymerization (MCP) of bis(1,6–heptadiyne) derivatives, and exhibited highly thermal and oxidative stability, low levels of lowest unoccupied molecular orbital (LUMO) energy levels (−4.3 ev), narrow band-gaps (1.70 ev), and regular ladder-like architecture [12]. However, it has poor solution processability and film-forming ability, because of the rigid PDI linkers, conjugated polyacetylene backbones, and strong aggregation, which severely restricted its application in photoelectric devices.

**Scheme 1:**
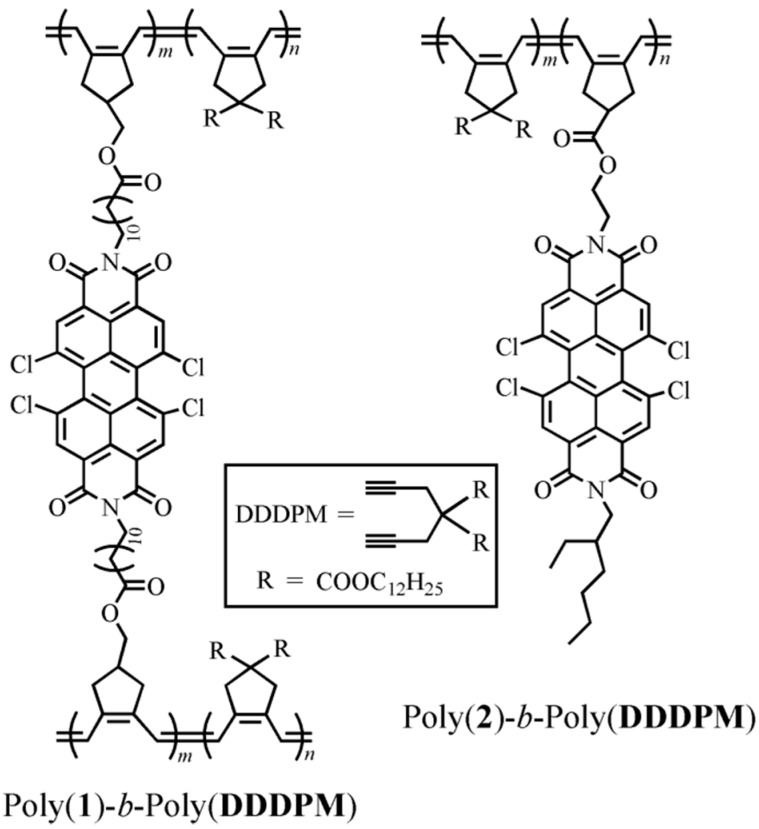
Structure of Poly(**1**)–*b*–Poly(**DDDPM**) and Poly(**2**)–*b*–Poly(**DDDPM**).

Investigations on PDI derivatives showed that increasing steric hindrance of 1,6,7,12-substitutes lead to more twisted perylene core and further reduced aggregation size [6,13]. This result suggested that breaking the coplanarity of the PDI core can improve the solution processability of organic molecules with a large π system [6]. Nevertheless, it is the prerequisite to the successful synthesis of polyacetylene-based ladderphanes, the spacing occupied by each of the poly(1,6–heptadiyne) unit (0.43 nm) would fit nicely into the PDI linker. Beyond traditional “twisting” methods, another effective strategy to achieve the polymeric ladderphanes with desired solution processability and film-forming ability is introducing aliphatic alkyl side chain. Hence, 1,6–heptadiyne monomer **DDDPM** with two long alkyl tails was synthesized, and then was copolymerized with PDI bridged bifunctionalized 1,6–heptadiyne monomer **1** (Scheme S1) and mono-functionalized 1,6–heptadiyne monomer **2** (Scheme S2), respectively, not only for improving the solution processability but also for tailoring the film morphology. Once synthesized, the expected copolymers (Scheme 1) should overcome the above mentioned two drawbacks and broaden the visible absorption region.

## 2. Results

For improving the solubility of poly(**1**), copolymerization of **1** and comonomer DDDPM bearing longer alkyl chain was supposed to be an attractive method to prepare soluble diblock copolymer, and therefore the copolymerization of **1** and didodecydipropargylmalonate (**DDDPM**) (Scheme S1 and Table 1) was carried out in THF at 30 °C with a lower [1]/[**DDDPM**]/[I] ratio of 10:80:1, yielding Poly(**1**)–*b*–Poly(**DDDPM**) with a narrow molecular weight distribution (PDI = 1.3) in 98% yield (Run 1). At a higher monomer loading, the higher [1]/[**DDDPM**]/[I] ratios of 20:80:1 and 40:80:1 (Runs 2~3) were attempted and expected to obtain higher molecular weight polymers, whereas the isolated polymer yields reduced to 76%, but there were two molecular weight values showing that some propagating carbenes discomposed when the polymer backbone increased to a certain size [14,15]. Similar results were found in the synthesis of Poly(**2**)–*b*–Poly(**DDDPM**), the optimal [2]/[**DDDPM**]/[I] ratio of 40:80:1, produced the polymer with a little wider PDI values (1.5) in 70% yield.

**Figure 1:**
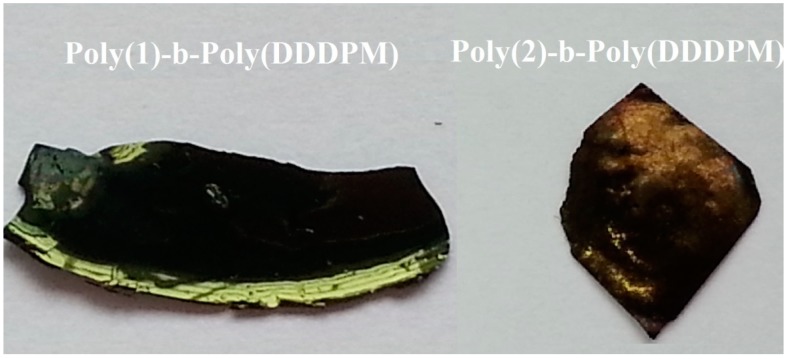
Picture of Poly(**1**)–*b*–Poly(**DDDPM**) and Poly(**2**)–*b*–Poly(**DDDPM**).

Both the resultant copolymers were easily soluble in common organic solvents (such as CHCl_3_, CH_2_Cl_2_, THF, chlorobenzene, and xylene), and had good film-forming properties. For example, when solutions of the copolymers in CH_2_Cl_2_ were added dropwise onto a slice of glass, and then after solvent evaporation, a black-green shiny film (Figure 1) were obtained, this observation illustrates the importance of introducing the soluble and flexible poly(**DDDPM**) block to poly(**1**), which indicating its great potential for solution processed organic devices and circuits. Similar results were also found for Poly(**2**)–*b*–Poly(**DDDPM**).

**Figure 2:**
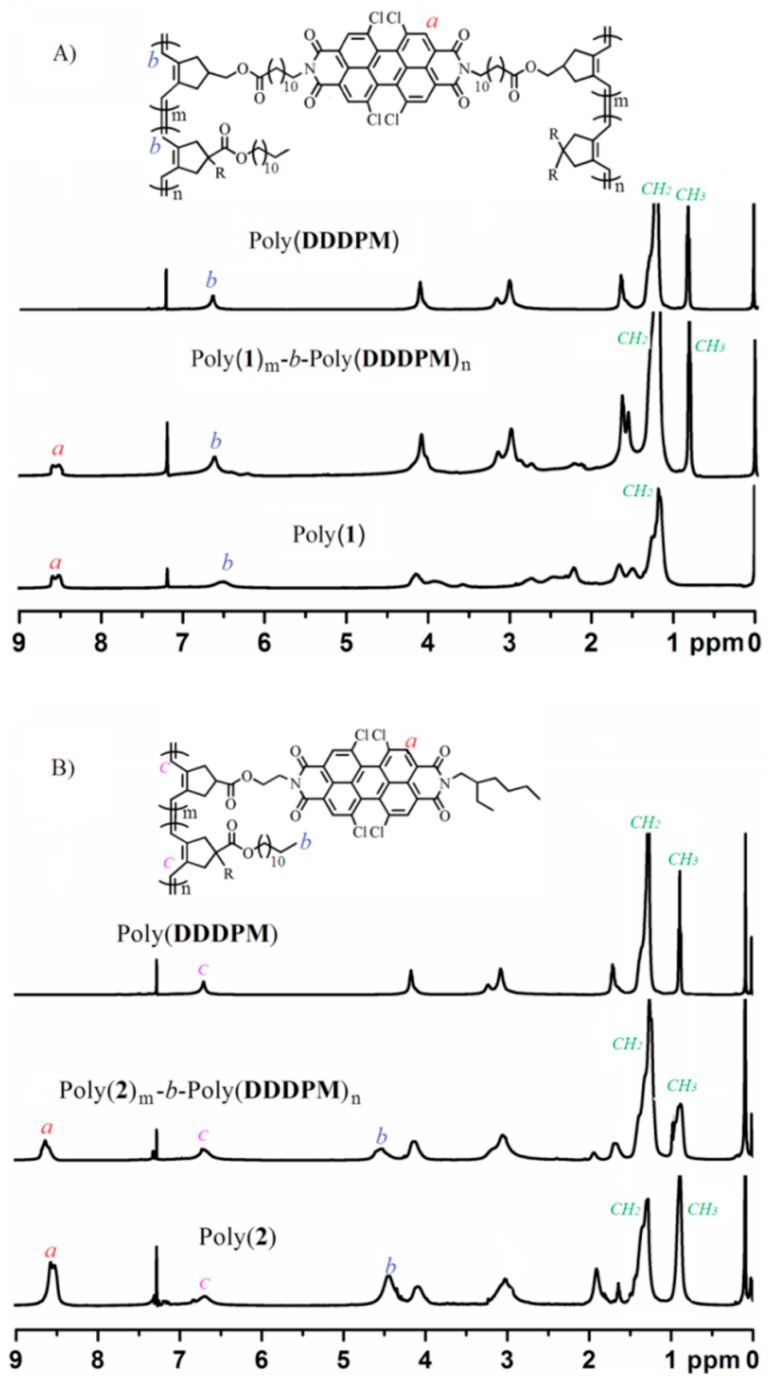
^1^H NMR spectrum of Poly(**1**)–*b*–Poly(**DDDPM**) (**A**) and Poly(**2**)–*b*–Poly(**DDDPM**) (**B**) in CDCl_3_.

^1^H NMR spectroscopy confirmed the expected structure of the diblock polymers, primarily displaying the characteristic signals regarding the Ar*Hs* and C*H_3_.* After copolymerization, the complete disappearance of the acetylenic proton at 2.02 ppm, and the emergence of symmetric broad peak at 6.5–7.0 ppm corresponding to the conjugated polyacetylene backbone indicate that a MCP reaction has occurred [16]. Moreover, two sets of new peaks at 8.65 and 0.90 ppm appeared (Figure 2A), which are exactly the characteristic peaks of perylene core on Poly(**1**) and terminal alkyl group on **DDDPM** (Figure S1). Coincidently, ratio of integral area of 8.65 and 0.90 ppm was 3:8, which matched ideally with the feeding ratio of [1]/[**DDDPM**]/[Ru–III] (20/80/1). Thus, this result confirmed the successful attachment of poly(**DDDPM**) to the poly (**1**) block, while, as can be seen from Figure 2B, the peaks at 8.65 and 4.56 ppm of Poly(**2**)-*b*-Poly(**DDDPM**) were derived from perylene core and methylene protons adjacent to nitrogen. There is no unique peak attributed to Poly(**DDDPM**). However, the integral ratio of methylene group and methyl groups on Poly(**2**)–*b*–Poly(**DDDPM**) changed from 4:3 to 8:3. This obvious increase was caused by the introduction of the second block Poly(**DDDPM**). Because there are two dodecyl groups on the **DDDPM** molecule, which contain many methylene group, thus, the increase of methylene group content in the copolymer is due to the introduction of **DDDPM** segments. The two blocks contain multiple methylene and methyl groups simultaneously, the content of the two blocks cannot be calculated by ^1^H NMR data. Meanwhile, it was found that GPC curves showed only one peak, and the molecular weight distributions of the diblock polymers were narrow, hence there were no homopolymer mixtures in the block polymer by GPC characterization (Figure S3).

**Figure 3:**
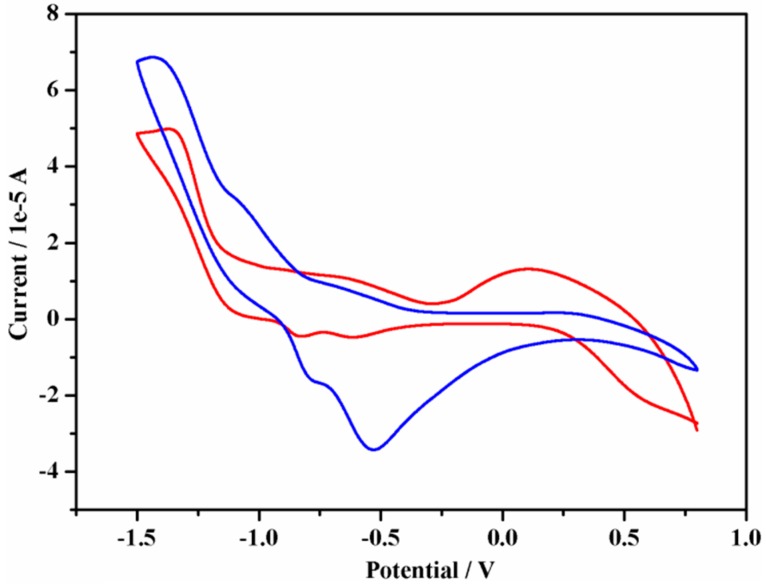
CV profiles of Poly(**1**)–*b*–Poly(**DDDPM**) (red line) and Poly(**2**)–*b*–Poly(**DDDPM**) (blue line).

To elucidate the influence of the conjugated poly(1,6–heptadiyne) backbone on the energy levels of the molecular orbitials, CV were applied to investigate the electrochemical performance of these copolymers (Figure 3), and the LUMO energy levels were calculated according to the onset reduction potentials ($E_{\mathrm{red}}^{\mathrm{onset}}$): *E*_LUMO_ = −(4.65 + $E_{\mathrm{red}}^{\mathrm{onset}}$) [17]. The LUMO values are −4.3 eV for Poly(**1**)–*b*–Poly(**DDDPM**), −4.0 eV for Poly(**2**)–*b*–Poly(**DDDPM**), lower than the well-studied PDI derivative (−3.8 eV) [18], and even comparable to that of fullerene derivatives (PCBM: −3.7 eV and C60: −4.5 eV) [19,20], indicating the higher electron-affinity and ambient stability [12].

**Figure 4:**
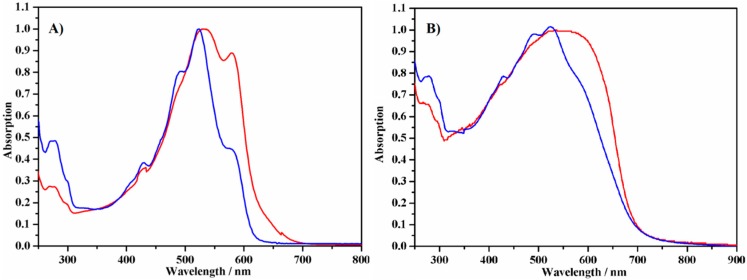
UV–vis spectra of Poly(**1**)–*b*–Poly(**DDDPM**) (red line) and Poly(**2**)–*b*–Poly(**DDDPM**) (blue line) in CH_2_Cl_2_ (**A**) and in film state (**B**).

Photophysical studies were characterized by UV–vis spectroscopy. CH_2_Cl_2_ was used as the solvent. Figure 4A shows characteristic absorption (400–600 nm) of PBI core, the PBI chromophore absorption maximum at 523 nm with a strongly pronounced vibronic fine structure is observed, which belongs to the electronic S_0_–S_1_ transition, with a transition dipole moment along molecular axis, and a second absorption band evolves at lower wavelengths (400–460 nm), which is attributed to the electronic S_0_–S_2_ with a transition dipole moment perpendicular to the long molecular axis [12]. Similar to other poly(1,6–heptadiyne) derivatives [21,22], Poly(**2**)–*b*–Poly(**DDDPM**) with flexible side chains also exhibiting relatively wide absorption with peaks in the range of 300–625 nm. While, Poly(**1**)–*b*–Poly(**DDDPM**) absorb above 550 nm with tailing near 700 nm. The peaks of Poly(**1**)–*b*–Poly(**DDDPM**) are red-shifted by about 75 nm in comparison with those of Poly(**2**)–*b*–Poly(DDDPM), owing to the rigidly ladderphane structure. This phenomenon suggested that distortion of the main chain happened which further caused the effective conjugated length decreased in Poly(**2**)–*b*–Poly(DDDPM). The optical properties of these copolymers were further characterized as thin film (Figure 4B), Relatively red-shifted (nearly 60 nm) were observed when compared with those in solution and the overall intensity are enhanced, indicated that there was π–π aggregation in film state, which is a necessary criterion for ensuring high electron mobilities [23]. According to the starting absorption wave-length of film state absorption, the values of *E*_g_ are calculated to be 1.55 eV for both of the copolymers.

**Figure 5:**
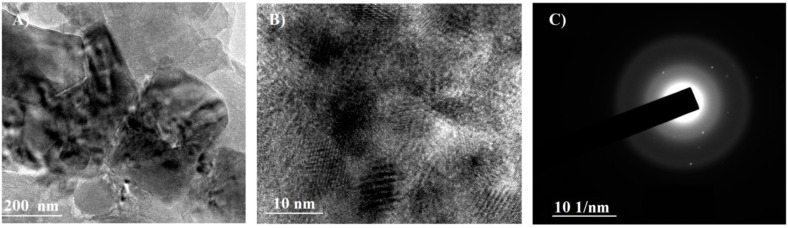
Transmission electron microscopy (TEM) images of Poly(**1**)-*b*-Poly(**DDDPM**) (**A**,**B**) and SAED pattern (**C**).

Double-stranded poly(1,6–heptadiyne) ladderphane are known to assemble nicely on copper mesh to give highly ordered two-dimensional patterns, presumably due to π–π attractions between terminal groups (vinyl and styryl groups) along the longitudinal axis of the polymers and van der Waals interactions between polymeric backbones. In a similar manner, Poly(**1**)–b–Poly(**DDDPM**) shows to be a layered polymer assembled on the copper mesh surface to form a ladderphane as revealed by its transmission electron microscopy (TEM) image (Figure 5A,B). The black strips aligned parallel to each other, suggesting that there is a strong interaction between molecules. In addition, the width for each strip was nearly 0.3 nm, indicating the aromatic PDI core would align perpendicular to the substrate orientation with respect to substrate surface, which would give layered structures. The relative selected area electron diffraction (SAED) pattern (Figure 5C) of Poly(**1**)–b–Poly(DDDPM) acquired during the TEM analysis further confirmed the highly ordered ladderphane structure. As expected, the structure of single-stranded Poly(**2**)–b–Poly(**DDDPM**) (Figure S4) was amorphous.

**Figure 6:**
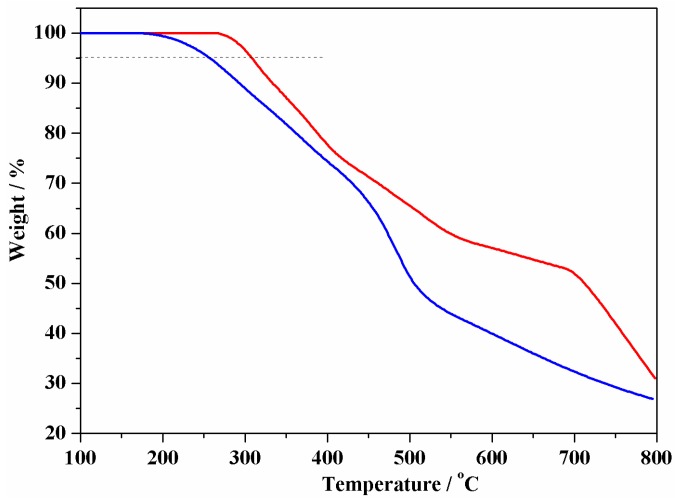
Thermogravimetric analysis (TGA) curves of copolymers Poly(**1**)–*b*–Poly(**DDDPM**) (red line) and Poly(**2**)–*b*–Poly(**DDDPM**) (blue line).

The effect of ladderphane structure on thermal degradation of the obtained diblock copolymers was studied by TGA. Figure 6 demonstrated that the weight loss of the copolymers had two interval steps, which mainly caused by the decomposition of diblock composition. When the temperature is higher than 325 °C for Poly(**1**)–*b*–Poly(**DDDPM**), the decrement just occurs mainly due to the decomposition of the side chains belonging to the copolymers, which is also important for the practical application of the polymers in devices and circuits, especially when they are used under high temperature. With the further increasing temperature, the main skeleton broke, resulting in a loss of nitrogen and hydrogen. Poly(**2**)–*b*–Poly(**DDDPM**) had poorer thermostability. The decomposition started at 235 °C, due to the single-stranded structure.

## 3. Materials and Methods

[1,3–Bis(2,4,6–trimethylphenyl)–2–imidazolidinylidene]dichloro(benzylidene)bis(3–bromopyridine)ruthenium(ii) (Grubbs third generation catalyst, Ru–III) and 1–dodecanol were obtained from Aldrich. Ethyl vinyl ether (stabilized with 0.1% *N*, *N*–diethylaniline) was purchased from Acros. 1–(3–dimethylaminopropyl)–3–ethylcarbodiimidehydrochloride(EDCI·HCl) and 4–Dimethylaminopyridine (DMAP) were purchased from Energy Chemical. Compound 2,2–bis(prop–2–yn–1–yl)malonic acid was synthesized according to the literature [24]. All reactions were carried out under dry nitrogen atmosphere using standard Schlenk-line techniques. Solvents were distilled over drying agents under nitrogen prior to use: dichloromethane (CH_2_Cl_2_) from calcium hydride, tetrahydrofuran (THF) from sodium. Polymerizations were carried out in Schlenk tubes. ^1^H (500 MHz) and ^13^C (125 MHz) NMR spectra were recorded using tetramethylsilane as an internal standard in CDCl_3_ on a Bruker DPX spectrometer. The HR–ESIMS was measured by a Bruker QTOF micromass spectrometer. UV–vis absorption spectra were measured on a Cary 60 spectrometer. Gel permeation chromatography (GPC) was used to calculate relative number average molecular weights (*M*_n_ and *M*_w_) and polydispersity index (PDI) equipped with a Waters 1515 Isocratic HPLC pump, a Waters 2414 refractive index detector, and a set of Waters Styragel columns (7.8 × 300 mm, 5 mm bead size; 10^3^, 10^4^, and 10^5^ Å pore size).Thermal gravimetric analysis (TGA) was performed using a SDTA851e/SF/1100 TGA Instrument under nitrogen flow at a heating rate of 10 °C /min from 50 to 800 °C. Cyclic voltammetry (CV) was carried out with an Autolab PGSTAT12 potentiostat from Eco Chemie coupled to an electrochemical cell with three electrodes. The scan rate was 100 mV/s. A glassy carbon electrode was used as a working electrode, a Pt wire as a counter electrode, and Ag/AgCl was used as the reference electrode. 0.1 M Bu_4_NPF_6_ of CH_3_CN solution was used as the supporting electrolyte, and Fc^+^/Fc was used as the reference.

### 3.1. Synthesis of Didodecydipropargylmalonate (DDDPM)

2,2–bis(prop–2–yn–1–yl)malonic acid (1.80 g, 10 mmol) was firstly dissolved in 50 mL of anhydrous CH_2_Cl_2_. To this solution, 1–dodecanol (4.10 g, 22 mmol), EDCI·HCl (4.20 g, 22 mmol) and DMAP (0.54 g, 4.4 mmol) were added under nitrogen atmosphere in ice-water bath and stirred for 2 h, then the reaction progress proceeded at room temperature and was monitored by TLC. After 6 days, the mixture was washed by dilute hydrochloric acid (5 × 30 mL), followed by water, and dried with anhydrous MgSO_4_. After filtration and removing the solvent, the crude product was purified by column chromatography on silica gel using 1:20 CH_2_Cl_2_/petroleum ether as eluent. DDDPM was obtained as a white waxy solid (4.03 g, yield 78%). ^1^H NMR (CDCl_3_, ppm): δ 4.20–4.12 (t, 4H, COOC*H*_2_), 2.65 (s, 4H, CHCC*H*_2_), 2.02 (s, 2H, C*H*CCH_2_), 1.45–1.7 (m, 40H, residual C*H*_2_), 0.94–0.80 (t, 6H, CH_2_C*H*_3_); ^13^C NMR (125 MHz, CDCl_3_, ppm): δ 178.28, 80.2, 70.5, 64.58, 50.6, 31.7, 29.6, 29.3,29.1, 25.8, 22.9, 22.4, 14.3; ESI–MS: Calcd. For C_33_H_56_O_4_Na [M + Na]^+^: 539.4176, Found: 539.4169.

### 3.2. Block Copolymerization

Typically, polymerization was carried out in a Schlenk tube under dry nitrogen atmosphere at 30 °C in THF for a preset time. Monomer **1** (100 mg, 0.08 mmol) and **Ru–III** (3.5 mg, 4 μmol) were stirred in 80 mL of THF at 30 °C. After it had been confirmed that **1** had disappeared by TLC (nearly 20 min), the 1 mL solution of the second block (**DDDPM**, 165 mg, 0.32 mmol) was added to the Schlenk tube, the reaction mixture was stirred at 30 °C for another 3 h. After being quenched by adding excess ethyl vinyl ether, the concentrated reaction mixture of Poly(**1**)–*b*–Poly(**DDDPM**) was precipitated to acetone, and the purple–black solid was washed with acetone till the filtrate was colorless, and then was dried in a vacuum oven at 40 °C to a constant weight.

The preparation of Poly(**2**)–*b*–Poly(**DDDPM**) was similar to that of Poly(**1**)–*b*–Poly(**DDDPM**), except for the initial concentration of monomer **2**. Monomer **2** (100 mg, 0.12 mmol) and **Ru–III** (5.3 mg, 6 μmol) were stirred in 1 mL of THF at 30 °C. After it had been confirmed that **2** had disappeared by TLC (nearly 2 h), the 1 mL solution of the second block (**DDDPM**, 247 mg, 0.48 mmol) was added to the Schlenk tube, and the reaction mixture was stirred at 30 °C for another 3 h.

## 4. Conclusions

In this study, diblock copolymer ladderphane containing PDI group was successfully synthesized via MCP reaction. By tailoring the proportion of the two blocks, novel poly(1,6–heptadiyne) derivatives with satisfactory solution processability and film-forming ability can be readily achieved without complicated and high-cost post-processing film formation techniques. This cannot be realized by small PDI compounds or oligomers. The *E*_g_ of Poly(**1**)–*b*–Poly(**DDDPM**) and Poly(**2**)–*b*–Poly(**DDDPM**) can even narrowed to 1.55 eV, and the LUMO energy lowered to −4.3 and −4.0 eV, respectively, which shows prospective application in solar cell and other devices. Based upon this study, further investigation of the application in photoelectric devices is still under way.

## Figures and Tables

**Table 1:** Gel permeation chromatography (GPC) data and photophysical properties of the resulted polymers.

Run	Sample	[M]/[I] *^a^*	*M*_n_*^c^* (kDa)	PDI *^d^*	Yield *^e^* (%)	*λ*_abs_*^f^* (nm)	*E*_g_*^g^* (eV)	LUMO *^h^* (eV)
1	Poly(**1**)-*b*-Poly(**DDDM**)	10:80:1	39.5	1.3	98	/		/
2	Poly(**1**)-*b*-Poly(**DDDM**)	20:80:1	48.8	1.5	95	800	1.55	−4.3
3	Poly(**1**)-*b*-Poly(**DDDM**)	40:80:1	34.6/72.5	1.4/1.5	76		/	/
4	Poly(**2**)-*b*-Poly(**DDDM**)	20:80:1	52.1	1.6	85		/	/
5	Poly(**2**)-*b*-Poly(**DDDM**)	40:80:1	66.0	1.5	70	800	1.55	−4.0
6	Poly(**2**)-*b*-Poly(**DDDM**)	60:80:1	40.2/83.4	1.5/1.6	41	/	/	/

Polymerization conditions: 30 °C, 3 h, THF, [M] = 1 × 10^−3^ mol/L. *^a^* The molar ratio of monomer to initiator. *^c^* Determined by GPC in THF relative to monodispersed polystyrene standards. *^d^* Degree of polymerization. *^e^* In chloroform solution. *^f^* In film state. *^g^* Bandgap of the film, *E*_g_ = 1240/*λ*_abs_. *^h^* Calculated from the onset reduction potentials of the polymers, *E*_LUMO_ = −(4.65 + $E_{\mathrm{red}}^{\mathrm{onset}}$).

## Supplementary Materials

Supplementary materials can be found at https://www.mdpi.com/1422-0067/20/20/5166/s1.

## Abbreviations

PDI	Perylene diimide
MCP	Metathesis cyclopolymerization
Ru-III	Grubbs third-generation catalyst
GPC	Gel Permeation Chromatography
CV	Cyclic voltammetry
TGA	Thermogravimetric analysis
LUMO	Lower unoccupied molecular orbital
DDDPM	Dipropargylmalonate
EDCI·HCl	1-(3-dimethylaminoprop-yl)-3-ethylcarbodiimidehydrochloride
DMAP	4-dimethylaminopyridine
GPC	Gel permeation chromatography
PCBM	[6,6]-phenyl-C61-butyric acid methyl ester
