# Supplementary material for: Functional Block Copolymers Carrying One Double-Stranded Ladderphane and One Single-Stranded Block in a Facile Metathesis Cyclopolymerization Procedure
Source: Int J Mol Sci. 2019 Oct 18;20(20):5166. doi: 10.3390/ijms20205166 (PMC6829535; doi:10.3390/ijms20205166)
Supplement: Supplementary file 1 [file ijms-20-05166-s001.pdf]

Functional Block Copolymers Carrying OneDouble-StrandedLadderphaneand One Single-Stranded Block in a Facile Metathesis Cyclopolymerization Procedure

Wei Song[1],*Yadi Li[1], Xunhu Liu[1], Zongyi Xu[1], Jianhua Wu [2,*], Liang Ding[1,*]

[1] Department of Polymer and Composite Material, School of Materials Engineering, Yancheng Institute of Technology, Yancheng, 224051, China
[2] Department of Materials, College of Physics, Mechanical and Electrical Engineering, Jishou University, Jishou, 416000, China
* Correspondence: sw121092@ycit.cn (W. Song); jianhuawu@jsu.edu.cn(J. H. Wu) and dl1984911@ycit.edu.cn (L. Ding); Tel.: +86 0515 88298872

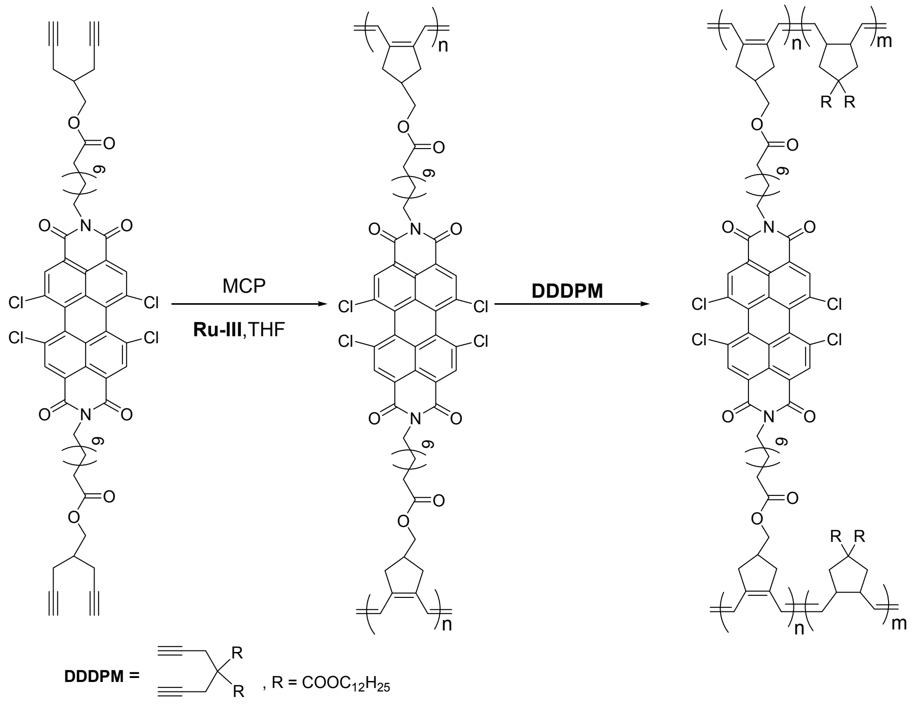

Scheme S1. Synthesis of Poly(**1**)-*b*-Poly(**DDDPM**)

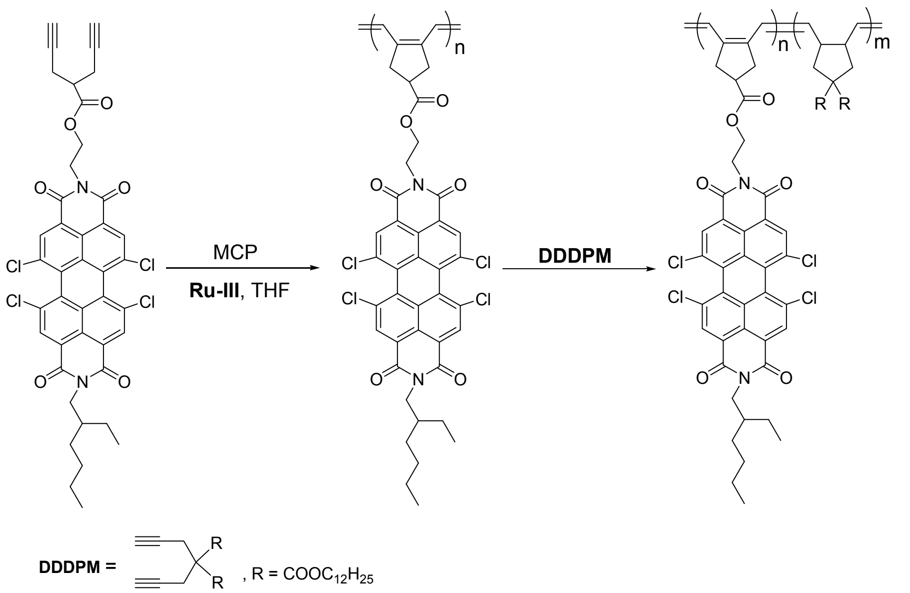

Scheme S2. Synthesis of Poly(**2**)-*b*-Poly(**DDDPM**).

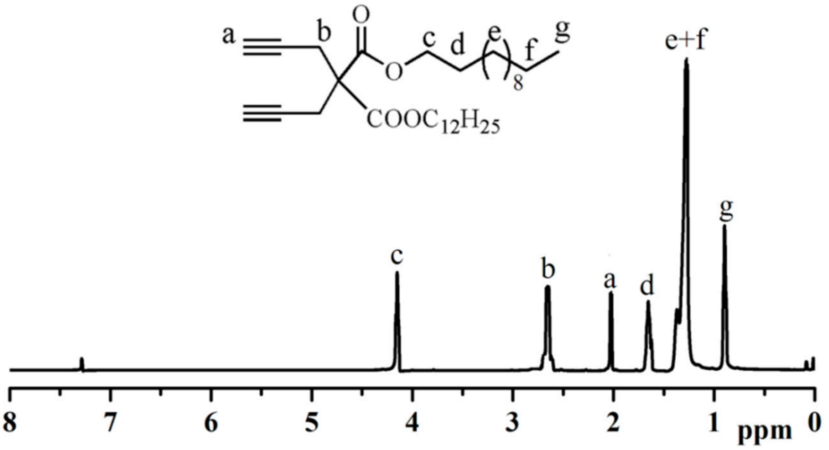

Figure S1. ^{1}H NMR spectrum of **DDDPM.**

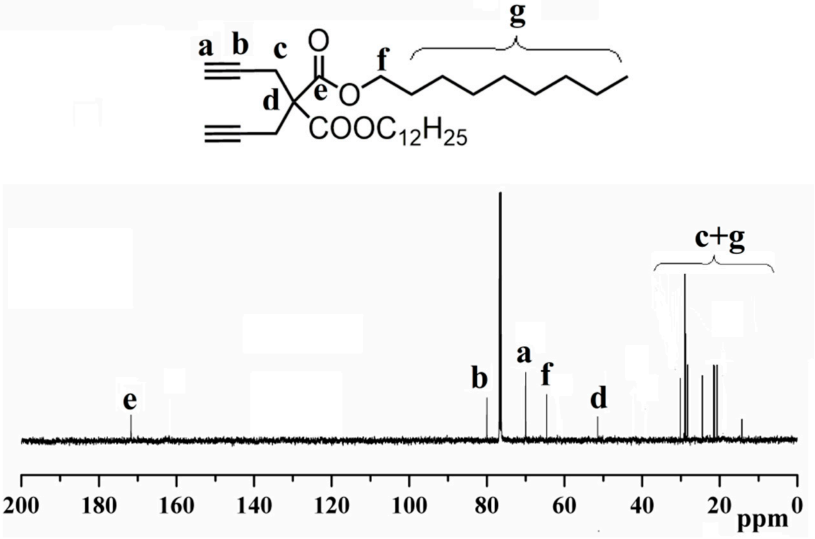

Figure S2. ^{1}H NMR spectrum of **DDDPM.**

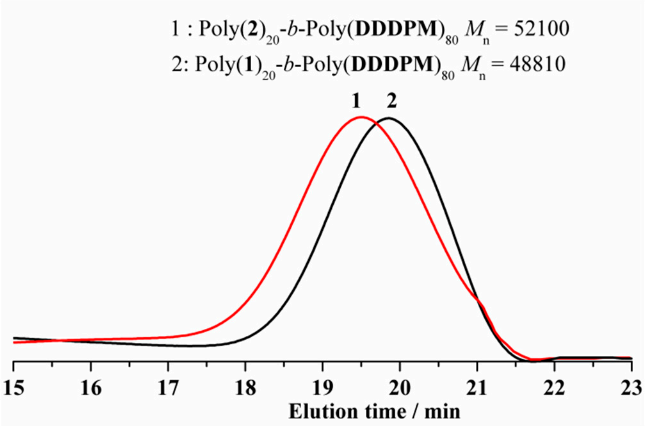

Figure S3. GPC curves of Poly(**1**)-*b*-Poly(**DDDPM**) and Poly(**2**)-*b*-Poly(**DDDPM**).

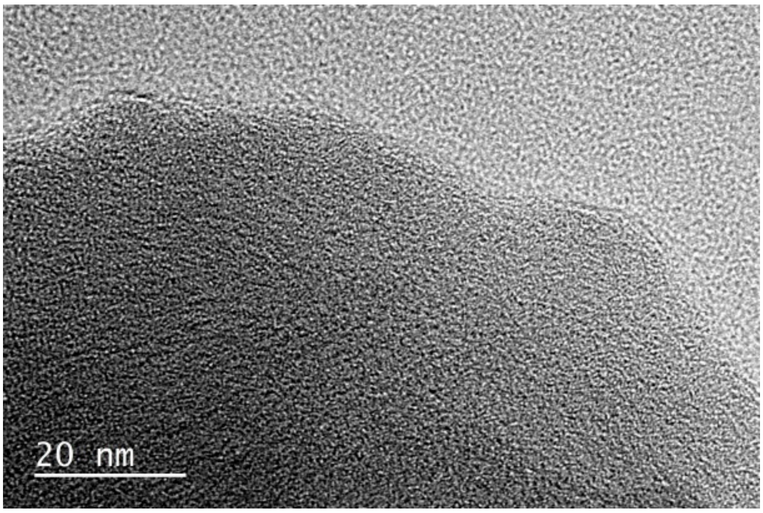

Figure S4. TEM image of Poly(**2**)-*b*-Poly(**DDDPM**).
